# Efficiency of a Preoperative Axillary Ultrasound and Fine-Needle Aspiration Cytology to Detect Patients with Extensive Axillary Lymph Node Involvement

**DOI:** 10.1371/journal.pone.0106640

**Published:** 2014-09-10

**Authors:** Isabella Castellano, Cristina Deambrogio, Francesca Muscarà, Luigi Chiusa, Giovanna Mariscotti, Riccardo Bussone, Guglielmo Gazzetta, Luigia Macrì, Paola Cassoni, Anna Sapino

**Affiliations:** 1 Department of Medical Sciences, University of Turin, Turin, Italy; 2 Istituto di Radiologia Diagnostica ed Interventistica, University of Turin, Città della Salute e della Scienza, Molinette Hospital, Turin, Italy; 3 Breast Surgery Department, Città della Salute e della Scienza, Sant’Anna Hospital, Turin, Italy; University of Utah School of Medicine, United States of America

## Abstract

**Background:**

Recent studies have demonstrated that axillary lymph node dissection (ALND) does not affect patient survival, even in those with one or two positive sentinel lymph nodes (SLNs). On the other hand, patients with 3 or more metastatic lymph nodes are eligible for chemotherapy. Therefore, it is crucial to identify *a priori* patients at risk of having a high number of metastatic axillary lymph nodes for their surgical and/or clinical management. Ultrasound (US) guided Fine-Needle Aspiration (FNA) has been proven to be a useful and highly specific method for detecting metastatic axillary lymph nodes. However, only one recent study has evaluated the efficiency of this method in identifying patients with high metastatic nodal involvement. Our aim was to validate US-guided FNA as a reliable method to discriminate *a priori* patients with >3 metastatic lymph nodes.

**Methods:**

A retrospective series of 1287 breast cancer patients who underwent a simultaneous preoperative breast and axillary US to stage their axilla was collected. A total of 365 patients, with either positive SLNs (278) or positive axillary lymph nodes detected via US-guided FNA (87), underwent ALND. In these two subgroups, we compared the number of metastatic lymph nodes in the axilla.

**Results:**

The number of metastatic axillary lymph nodes in patients who underwent US-guided FNA was significantly higher (63% had >3 metastatic lymph nodes) than that in patients with SLNs positive for micro- or macrometastases (3% and 27%, respectively) (*P*<0.001, *χ* ^2^ = 117.897).

**Conclusions:**

Preoperative axillary US-guided FNA could act as a reliable tool in identifying breast cancer patients with extensive nodal involvement.

## Introduction

For years, the sentinel lymph node (SLN) biopsy has been considered the standard technique in staging axillary lymph nodes in breast cancer patients [1]. Traditional protocols indicate that breast cancer patients with positive SLNs require a thorough examination of their axillary lymph nodes, while patients with negative SLNs can skip axillary lymph node dissection (ALND). However, the optimal management of the axilla is a matter of discussion again, following the results of a trial by Giuliano et al. [2]. This study did not detect any differences in survival among patients with T1–T2 breast cancer, with one or two positive SLNs, treated with breast conservation and systemic therapy alone or with ALND. Other studies [3], [4] have reported that only 30% of patients with positive SLNs showed involvement of other axillary lymph nodes and that axillary recurrence in patients not treated with ALND was uncommon.

On the other hand, the status of axillary lymph nodes remains an important prognostic index of overall and disease-free survival, and the number of metastatic nodes is considered by oncologists when deciding whether to administer chemotherapy, mainly in patients with estrogen receptor (ER)-positive cancer [5]. In particular, the last Consensus Meeting in St. Gallen recommended the use of chemotherapy in patients with more than 3 metastatic axillary lymph nodes [6].

Therefore, for both the surgical and oncological management of patients, it is critical to identify *a priori* those eligible for ALND. To this end, several nomograms have been proposed [7], [8].

Axillary ultrasonography (US) guided Fine-Needle Aspiration cytology (FNA) of suspicious lymph nodes is recognised as an optimal preoperative procedure for identifying patients eligible for ALND [9]–[12]. In 2003, we published one of the first studies demonstrating that US-guided FNA of suspicious axillary lymph nodes could reliably be introduced as a preoperative procedure, to address the surgical management of the axilla [9]. However, only one recent study has evaluated the efficiency of US-guided FNA to identify patients with high metastatic nodal involvement (>3 metastatic lymph nodes) [13]. Taking into account the results of van Wely et al. [13], the present study aimed to validate US-guided FNA as a reliable method that could be introduced into routine practice to discriminate *a priori* patients with extensive metastatic nodal involvement from those at low risk.

## Methods

A retrospective case-series of 1287 invasive breast cancer patients, operated between January 2004 and December 2012, was collected (excluding patients on neoadjuvant therapy). This cohort underwent simultaneous preoperative breast and axillary US to stage the axilla. The criteria for defining a lymph node suspected of containing metastases by US were (1) the loss of lymph node shape; (2) a rounded appearance; (3) an increased cortical thickening; (4) an echo-poor central hilus and (5) an eccentricity of the nodal cortex.

In our Breast Unit, the protocol for the management of patients with suspicious lymph nodes after a US examination (independent of the number of suspect lymph nodes) is to perform a US-guided FNA procedure, before proceeding to surgery. The axillary FNA is performed on a single lymph node, using a 22-Gauge needle. In case of multiple suspect lymph nodes, the easiest to target is aspirated. The cytological material is smeared and immediately fixed in methanol and stained with hematoxylin and eosin. The pathologist, present on site, gives a diagnosis based on the presence or absence of cancer cells in the smear. Hypocellular smears are considered inadequate and additional FNAs are performed depending on the feasibility of the aspiration procedure.

**Figure 1:**
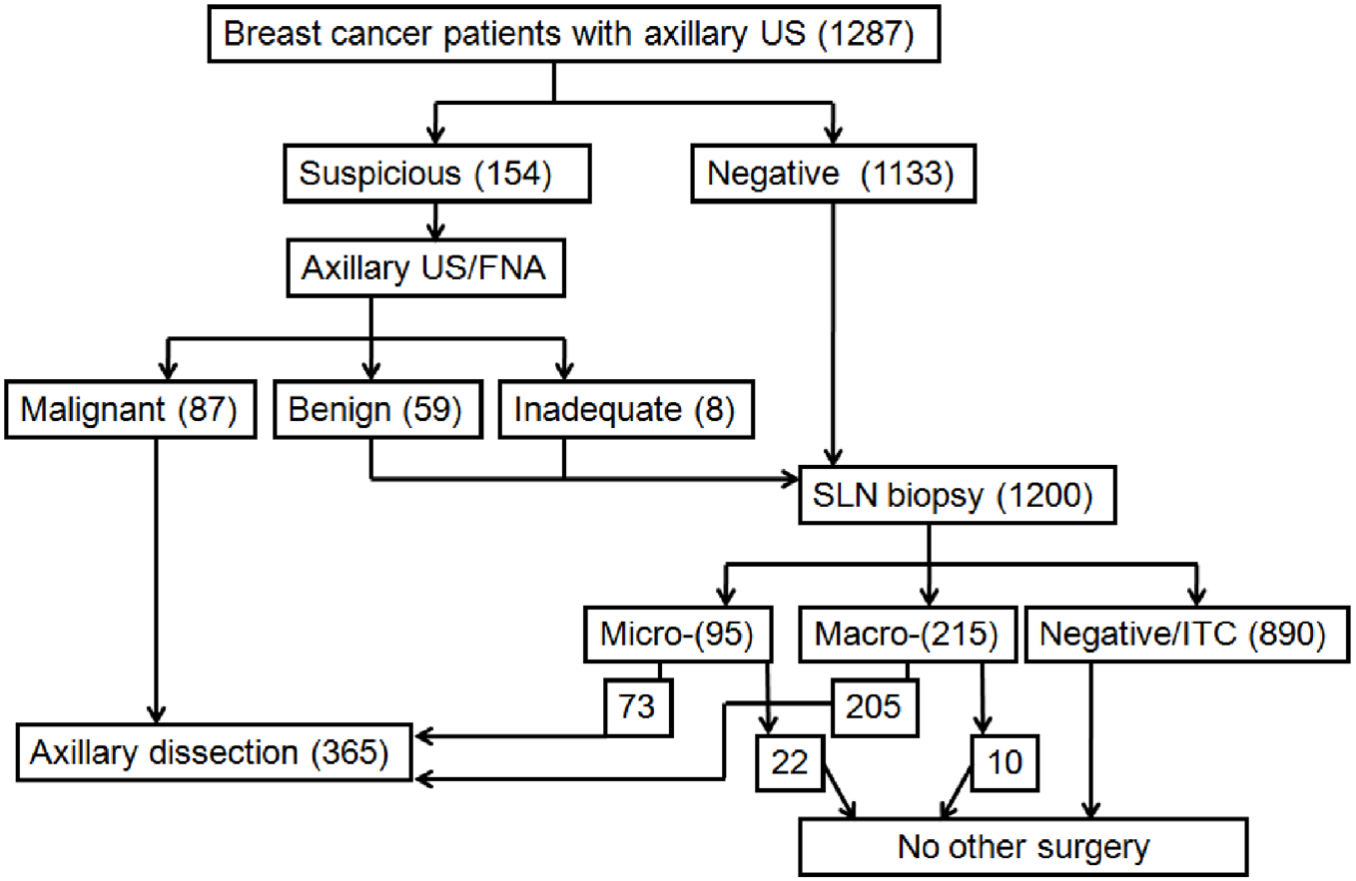
Axillary lymph nodes management in breast cancer patients US: ultrasound; FNA: fine needle aspiration; SLN: sentinel lymph node; ITC: isolated tumour cells.

Patients with malignant axillary lymph nodes following US-guided FNA bypass the SLN biopsy and undergo an ALND, whereas those with normal axillary lymph nodes or benign/inadequate FNA cytology receive a SLN biopsy followed by an ALND if their SLN is positive (Figure 1). The SLN histopathological assessment is performed following the regional guidelines [14] and the diagnosis is reported based on the TNM classification [15].

If an ALND is performed, the number of excised lymph nodes and the number of lymph nodes positive for metastases are reported in the histopathological diagnosis.

For the purposes of the study, patient age, tumour histotype, grade, size, vascular invasion, hormone receptor expression, Ki67 index value and HER2 status (Table 1) were recorded in a dedicated database.

**Table 1:** Clinical and histopathological characteristics of 1287 breast cancer patients who underwent axillary US examination.

	Number of cases	%
**Age (years)**		
<40	47	3.6
40–49	185	14.3
50–69	753	58.5
≥70	302	23.4
**Histotype**		
Ductal	887	69
Lobular	249	19.3
Others	151	11.7
**Grading**		
1	322	24.8
2	667	51.8
3	298	23
**pT**		
1a	35	2.7
1b	236	18.3
1c	691	53.6
2	291	22.6
3	11	0.08
4	23	0.17
**SLN**		
Negative+ITC	890	68.6
Micrometastasis	95	7.3
Macrometastasis	215	16.7
Not done	87	6.7
**Vascular invasion**		
Absent	875	68
Present	412	32
**Estrogen Receptor**		
0%	148	11.5
≥1%	1139	88.5
**Progesterone Receptor**		
0%	231	18
≥1%	1056	82
**Ki67**		
<14%	526	40.8
≥14%	761	59.2
**HER2**		
Negative	1115	86.6
Positive	172	13.4

Legend: SLN: Sentinel Lymph Node; ITC: Isolated Tumour Cells.

All clinical investigations have been conducted according to the principles expressed in the Declaration of Helsinki.

The study was approved by the ethic institutional review board for “Biobanking and use of human tissue for experimental studies” of the Pathology Services of the Azienda Ospedaliera Città della Salute e della Scienza di Torino. Written informed consent was obtained. Patient records were anonymized and de-identified prior to analysis.

The data were analysed using the SPSS version 19 software (SPSS, Inc., Chicago, IL. USA). Statistical significance was set at *P*<0.05. Statistical analyses were performed using the chi-squared test. A multivariate survival analysis was performed using the Cox model. The concordance between an axillary US, an axillary FNA and a SLN biopsy and the axillary lymph node status after an ALND was estimated using the Cohen’s κ statistic. The sensitivity, specificity, positive predictive values (PPV) and negative predictive values (NPV) of the combined axillary US-guided FNA and of US alone were calculated.

## Results

The clinical and pathological characteristics of 1287 patients with breast cancer who underwent an axillary US examination are described in Table 1.

In 154 (12%) patients, the US examination detected at least 1 suspicious lymph node, which was subjected to a US-guided FNA. Of these cytological samples, 87 (57%) were diagnosed as malignant (presence of cancer cells) and 59 (38%) as benign (absence of cancer cells). Eight (5%) FNA specimens were inadequate for diagnosis (Figure 1). ALND was performed in the 87 patients with malignant FNA, while the others underwent a SLN biopsy. Altogether, the SLN surgical procedure was performed in 1200 patients (67 diagnosed as benign or having inadequate axillary lymph nodes following US-guided FNA and 1133 displaying no axillary involvement following a US) (Figure 1). The mean number of SLNs excised was 1.2. The final histopathological diagnoses of SLNs reported 215 (17.9%) patients with macrometastases, 95 (7.9%) with micrometastases, 36 (3%) with isolated tumour cells and 854 (71%) as negative. An ALND was performed in 278 patients (205 positive for macrometastases and 73 positive for micrometastases). The 10 patients with SLNs positive for macrometastases rescued from the ALND were older than 80 years of age (Figure 1).

### Performance of combined axillary US-guided FNA and SLN

All patients with positive axillary lymph nodes following US-guided FNA showed metastatic disease in the ALND (Table 2).

**Table 2:** Metastatic involvement of the axilla in 154 breast cancer patients that underwent US-guided FNA.

US-guided FNA 154 (%)	Metastatic involvement of the axilla		Cohen’s κ statistic
	Yes (%)	No (%)	
Malignant 87 (56.5)	87 (100)	0	0.77
Benign/Inadequate 67 (43.5)	17 (25)	50 (75)	

Legend: US: Ultrasonography; FNA: Fine-Needle Aspiration cytology; ALND: Axillary Lymph Node Dissection; SLN: Sentinel Lymph Node.

The Cohen’s κ statistic for the concordance between the cytological results and histological axillary assessment following ALND was 0.77 (Table 2).

With the aim to evaluate the performance of US alone in detecting metastatic lymph nodes in the axilla, we focused on 67 cases with benign or inadequate US-guided FNA, which underwent SLN biopsy. As shown in Table 3, we found 17 cases identified as suspect by US and benign or inadequate by FNA, but ultimately with metastatic SLN. None of them had an extracapsular extension. In particular, 8 macro- and 4 micrometastatic SLNs were observed in 12 out of 59 patients with benign axillary FNA and 3 macro- and 2 micrometastatic SLNs in 5 out of 8 patients with inadequate FNA samples. Thus, the US alone had a sensitivity of 26.2%, a specificity of 94.3%, a PPV 67.5% of and a NPV of 74.1%. On the contrary US-guided FNA reached 83.7% sensitivity, 100% specificity, 100% PPV and 74.6% NPV.

**Table 3:** Relationship between US of the axilla with a suspect result, not confirmed by FNA and metastases in SLN.

US-Suspect/FNA benign/inadequate	SLN positive	SLN negative	Total
FNA Benign	12	47	59
FNA Inadequate	5	3	8
**Total**	17	50	67

Legend: US: Ultrasonography; FNA: Fine-Needle Aspiration cytology; SLN: Sentinel Lymph Node.

**Figure 2:**
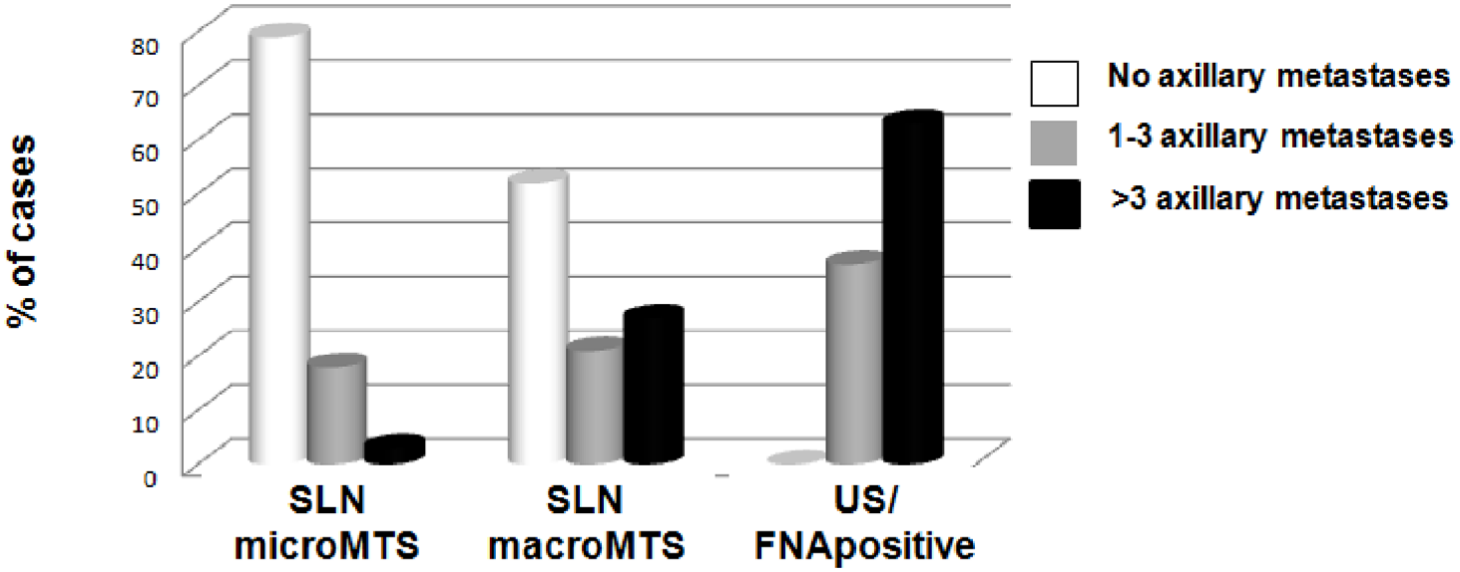
Number of axillary metastatic lymph nodes in breast cancer patients with positive (micro- and macrometastases) sentinel lymph nodes (SLNs) and in patients with positive ultrasound (US) guided fine needle aspiration cytology (FNA). The percentage of cases with >3 axillary metastatic lymph nodes is higher in patients with positive US-guided FNA cytology than in patients with metastatic SLNs.

In addition, we analysed the number of metastatic lymph nodes detected after ALND, performed following the detection of either a malignant US-guided FNA cytology or of a positive SLN. In the first set of patients, 63% had >3 metastatic lymph nodes, whereas only 20% of patients with metastatic SLN showed metastases in >3 lymph nodes (Table 4). In particular, as shown in Figure 2, only 2.7% of the patients with SLN micrometastases and 26.8% with SLN macrometastases had metastases in >3 lymph nodes (*P*<0.001, *χ* ^2^ = 117.897). Moreover, of the 17 patients with negative or inadequate FNA and positive SLNs, only 1 (5.8%) showed >3 metastatic lymph nodes; on the other hand 79.4% of the patients with micrometastases and 52% of the patients with macrometastases in the SLNs did not show any additional metastatic lymph nodes after ALND (Figure 2).

**Table 4:** Status of axillary lymph nodes in 365 breast cancer patients with positive US-guided FNA or positive SLN.

	No metastases in the ALND	Metastases in the ALND	
		1–3 LN*	>3 LN*
**Malignant US-guided FNA (87)**	0	32 (36.7%)	55 (63.2%)
**SLN Micro-and macroMTS (278)**	165 (59.3%)	56 (20.2%)	57 (20.5%)

Legend: SLN: Sentinel Lymph Node; US: Ultrasonography; FNA: Fine-Needle Aspiration cytology. MTS: metastases; ALND: axillary lymph node dissection; LN: lymph node.

*Including SLN.

In univariate analyses, malignant axillary lymph nodes following US-guided FNA correlated with larger tumour size, higher tumour grade, presence of vascular invasion, absence of progesterone receptor expression, HER2 positivity, and Ki67>14% (Table 5). However, in multivariate analyses only the pT-status maintained its statistical significance (P = 0.01, OR 0.127, confidence interval 0.224–0.030).

**Table 5:** Clinical and histological characteristics of patients stratified on the basis of axillary US-guided FNA results.

	Malignant US guided/FNA	Benign US guided/FNA	Inadequate US guided/FNA	NO US guided/FNA	P value (X^2^)
	87 (%)	59 (%)	8 (%)	1133 (%)	
**Age (years)**					
<40	4 (4.5)	1 (1.6)	1 (12.5)	41 (3.6)	
40–49	18 (20.6)	9 (15.2)	1 (12.5)	157 (13.8)	*Ns*
50–69	45 (51.7)	33 (56)	4 (50)	671 (59.2)	
≥70	20 (23)	16 (27.1)	2 (25)	264 (23.3)	
**pT**					
pT1	27 (31)	45 (76.2)	5 (62.5)	885 (78.1)	
pT2	44 (50.5)	12 (20.3)	3 (37.5)	232 (20.4)	<0.001
pT3	6 (6.8)	0	0	5 (0.4)	(25.385)
pT4	10 (11.5)	2 (3.3)	0	11 (1)	
**Histotype**					
Ductal	65 (74.7)	41 (70)	8 (100)	773 (68.2)	
Lobular	13 (15)	10 (17)	0	226 (20)	*Ns*
Other	9 (10.3)	8 (13.5)	0	134 (11.8)	
**Grade**					
1	8 (9.1)	19 (32.2)	2 (25)	293 (26)	
2	37 (42.5)	25 (42.3)	4 (50)	601 (53)	<0.001
3	42 (48.3)	15 (25.4)	2 (25)	239 (21)	(36.326)
**VI**					
Absent	21 (24)	43 (73)	4 (50)	807 (71.2)	<0.001
Present	66 (76)	16 (27)	4 (50)	326 (28.8)	(84.161)
**ER**					
0	13 (15)	7 (12)	0	128 (10.3)	*Ns*
>1%	74 (85)	52 (88)	8 (100)	1005 (88.7)	
**PR**					
0	27 (31)	10 (17)	0	194 (17)	<0.001
>1%	60 (69)	49 (83)	8 (100)	939 (83)	(10.849)
**HER2**					
Negative	62 (71)	52 (88)	7 (87)	994 (88)	<0.001
Positive	25 (29)	7 (12)	1 (3)	139 (12)	(19.049)
**Ki67**					
<14%	20 (23)	24 (40.6)	2 (25)	480 (42.3)	<0.001
≥14%	67 (77)	35 (59.3)	6 (85)	653 (57.7)	(12.346)

Legend: US/FNA: US-guided FNA; US: Ultrasonography; FNA: Fine-Needle Aspiration cytology; VI: vascular invasion; ER: Estrogen Receptor; PR: Progesterone Receptor.

## Discussion

With the present study, we were able to validate the results recently published by van Wely et al. [13] showing that the pre-surgical US-guided FNA of axillary lymph nodes is a reliable method with which to select patients at risk of high metastatic involvement.

Several studies have been shown that the occurrence of metastases in the lymph nodes of the axilla, even after one positive SLN, could be a rare event [16], [17], and nomograms have been constructed to save patients from ALNDs, using parameters mainly derived *a posteriori*, from the histopathological analysis of a tumour specimen (size, grade, vascular invasion) and/or SLN metastases [7], [8], [18]–[21]. However, Wo et al. [22] demonstrated that even patients with small tumours (<0.5 cm in diameter) could have several metastatic lymph nodes and that these patients have a higher breast cancer-specific mortality than those with larger tumours. As expected, our results confirmed that malignant axillary lymph nodes, following US-guided FNA, correlated with large tumour size (>20 mm) [23], [24]; nevertheless, 31% of the tumours were classified as pT1 (≤20 mm), and of these, 3 out of 27 (11.5%) were less than 1 cm. Moreover, in the same subgroup of pT1 cases with malignant US-guided FNA, 9 patients out to 27 showed 1 to 3 metastatic lymph nodes and the 18 cases had more than 3 metastatic lymph nodes.

Taking these parameters into account, it is evident that validating the reliability of the proposed method to identify *a priori* (before any type of surgery) the patients at high risk of axillary metastases is of both clinical and surgical relevance.

Pre-surgical examination of axillary lymph nodes by US itself has good specificity. In one study, the detection of suspicious lymph nodes by US was correlated with a high number of metastatic axillary lymph nodes [11]. However, US alone is characterised by low sensitivity in defining the presence of metastases [12], [25]. The additional cytological assessment of axillary lymph nodes using US-guided FNA improves sensitivity and reaches an absolute specificity of 100% for the diagnosis of metastases [9], [26]. In particular, we showed that while >50% of patients with a SLN affected by macrometastases did not show additional metastases in the axilla, none of the patients with a positive US-guided FNA had negative axillary lymph nodes. In addition, the US-guided FNA method is able to better identify breast cancer patients presenting with >3 metastatic lymph nodes (63% US-guided FNA *versus* 26.8% of SLN macrometastases and 2.7% of SLN micrometastases) than the SLN method.

Therefore, the take-home message of the present findings, which confirms the results of van Wely et al. [13], is that positive US-guided FNA cytology performed on a single lymph node could be a marker for the possible involvement of >1 axillary lymph node, particularly in large tumours, and thus reliably leads to one-step ALND.
